# Supplementary material for: Rashba Torque Driven Domain Wall Motion in Magnetic Helices
Source: Sci Rep. 2016 Mar 24;6:23316. doi: 10.1038/srep23316 (PMC4806324; doi:10.1038/srep23316)
Supplement: Supplementary Information [file srep23316-s1.pdf]

Supplementary to

Rashba Torque Driven Domain Wall Motion in Magnetic Helices

Oleksandr V. Pylypovskyi[1,*], Denis D. Sheka[1,†], Volodymyr P. Kravchuk[2,‡], Kostiantyn V. Yershov[2,3,§], Denys Makarov[4,5,¶], and Yuri Gaididei[2,]**

[1]Taras Shevchenko National University of Kyiv, 01601 Kyiv, Ukraine
[2]Bogolyubov Institute for Theoretical Physics of the National Academy of Sciences of Ukraine, 03680 Kyiv, Ukraine
[3]National University of "Kyiv-Mohyla Academy", 04655 Kyiv, Ukraine
[4]Helmholtz-Zentrum Dresden-Rossendorf e. V., Institute of Ion Beam Physics and Materials Research, 01328 Dresden, Germany
[5]Institute for Integrative Nanosciences, IFW Dresden, 01069 Dresden, Germany
[*]engraver@univ.net.ua
[†]sheka@univ.net.ua
[‡]vkravchuk@bitp.kiev.ua
[§]yershov@bitp.kiev.ua
[¶]d.makarov@hzdr.de
[**]ybg@bitp.kiev.ua

ABSTRACT

The supplementary information provides details on analytical calculations of main aspect of the magnetization statics and dynamics of the transversal domain wall in a helix wire.

1 The model

Let us consider a curvilinear magnetic wire, which can be modelled by the 3D curved $\boldsymbol{\gamma} \subset \mathbb{R}^3$. We describe the magnetic properties of the wire using assumptions of classical ferromagnet with uniaxial anisotropy directed along the wire. The easy-tangential anisotropy in a curved magnet is spatially dependent. In order to describe the magnetization distribution in such systems it is convenient to use a curvilinear Frenet–Serret (TNB) parametrization of the curve $\boldsymbol{\gamma}$:

$$\boldsymbol{e}_{\mathrm{T}} = \partial_s \boldsymbol{\gamma}, \qquad \boldsymbol{e}_{\mathrm{N}} = \frac{\partial_s \boldsymbol{e}_{\mathrm{T}}}{|\partial_s \boldsymbol{e}_{\mathrm{T}}|}, \qquad \boldsymbol{e}_{\mathrm{B}} = \boldsymbol{e}_{\mathrm{T}} \times \boldsymbol{e}_{\mathrm{N}}$$

with $\boldsymbol{e}_{\mathrm{T}}$ being the tangent, $\boldsymbol{e}_{\mathrm{N}}$ being the normal, and $\boldsymbol{e}_{\mathrm{B}}$ being the binormal to $\boldsymbol{\gamma}$ and s being the arc length. In particular, we use TNB parametrization of the magnetization unit vector,

$$\boldsymbol{m} = \left(m_{\mathrm{T}}, m_{\mathrm{N}}, m_{\mathrm{B}}\right)^T \tag{S1}$$

with the curvilinear components m_α. Here and below Greek indices α, β numerate curvilinear coordinates (TNB-coordinates) and curvilinear components of vector fields. For an arbitrary thin wire the energy can be presented as follows[1]

$$\begin{aligned}
&E = K^{\mathrm{eff}} S \int \mathcal{E} \mathrm{d}s, \qquad \mathcal{E} = \mathcal{E}_{\mathrm{ex}} + \mathcal{E}_{\mathrm{an}},\\
&\mathcal{E}_{\mathrm{ex}} = \mathcal{E}_{\mathrm{ex}}^0 + \mathcal{E}_{\mathrm{ex}}^{\mathrm{D}} + \mathcal{E}_{\mathrm{ex}}^{\mathrm{A}}, \quad \mathcal{E}_{\mathrm{ex}}^0 = \left|\boldsymbol{m}'\right|^2,\\
&\mathcal{E}_{\mathrm{ex}}^{\mathrm{D}} = \mathscr{F}_{\alpha\beta} \left(m_\alpha m_\beta' - m_\alpha' m_\beta\right), \quad \mathcal{E}_{\mathrm{ex}}^{\mathrm{A}} = \mathscr{K}_{\alpha\beta} m_\alpha m_\beta,\\
&\mathcal{E}_{\mathrm{an}} = -m_{\mathrm{T}}^2,
\end{aligned} \tag{S2}$$

where the Einstein notation is used for summation, $K^{\mathrm{eff}} = K + \pi M_s^2$, where the positive parameter K is a magnetocrystalline anisotropy constant of easy-tangential type, the term πM_s^2 comes from the magnetostatic contribution[2–4] and S is the cross-section area. Here and below the prime denotes the derivative with respect to the dimensionless coordinate $u = s/\ell$ with

$\ell = \sqrt{A/K^{\text{eff}}}$ being a magnetic length (A is an exchange constant). The first term in the exchange energy $\mathcal{E}_{\text{ex}}^{0}$ describes the common isotropic part of exchange expression which has formally the same form as for the straight wire. The second term $\mathcal{E}_{\text{ex}}^{\text{D}}$ in the exchange energy functional is a curvature induced effective Dzyaloshinskii-Moriya interaction (DMI), which is linear with respect to curvature and torsion. The tensor of coefficients of such interaction is the dimensionless Frenet–Serret tensor[1]

$$\left\|\mathscr{F}_{\alpha\beta}\right\| = \begin{pmatrix} 0 & \varkappa & 0 \\ -\varkappa & 0 & \sigma \\ 0 & -\sigma & 0 \end{pmatrix}.$$

Here $\varkappa = \kappa\ell$ and $\sigma = \tau\ell$ are the dimensionless curvature and torsion, respectively, with κ being the curvature and τ being the torsion. The term $\mathcal{E}_{\text{ex}}^{\text{A}}$ describes an effective anisotropy interaction, where the components of the tensor $\mathscr{K}_{\alpha\beta} = \mathscr{F}_{\alpha\nu}\mathscr{F}_{\beta\nu}$ are bilinear with respect to the curvature and the torsion,

$$\left\|\mathscr{K}_{\alpha\beta}\right\| = \begin{pmatrix} \varkappa^2 & 0 & -\varkappa\sigma \\ 0 & \varkappa^2+\sigma^2 & 0 \\ -\varkappa\sigma & 0 & \sigma^2 \end{pmatrix}.$$

The energy of effective anisotropy

$$\mathcal{E}_{\text{eff}}^{\text{A}} = \mathcal{E}_{\text{an}} + \mathcal{E}_{\text{ex}}^{\text{A}} = \mathscr{K}_{\alpha\beta}^{\text{eff}} m_\alpha m_\beta, \quad \mathscr{K}_{\alpha\beta}^{\text{eff}} = \mathscr{K}_{\alpha\beta} - \delta_{\alpha,1}\delta_{\beta,1}$$

has a form, typical for biaxial magnets. The tensor of effective anisotropy coefficients $\mathcal{K}_{\alpha\beta}^{\text{eff}}$ has non–diagonal components. This means that the homogeneous magnetization structure is not oriented along the TNB basis. One can easily diagonalize it, by using a unitary transformation (rotation in a local rectifying plane) of the vector $\boldsymbol{m}$ (S1)

$$\boldsymbol{m} = U\widetilde{\boldsymbol{m}}, \quad \widetilde{\boldsymbol{m}} = U^{-1}\boldsymbol{m}, \quad \widetilde{\boldsymbol{m}} = \left(m_1, m_2, m_3\right)^T \qquad U = \begin{pmatrix} \cos\psi & 0 & -\sin\psi \\ 0 & 1 & 0 \\ \sin\psi & 0 & \cos\psi \end{pmatrix}.$$

By choosing the rotation angle ψ as follows

$$\psi = \arctan\frac{\sigma\varkappa}{\mathcal{K}_0}, \qquad \mathcal{K}_0 = \frac{1+\sigma^2-\varkappa^2+\mathcal{K}_1}{2}, \quad \mathcal{K}_1 = \sqrt{(1-\varkappa^2+\sigma^2)^2+4\varkappa^2\sigma^2}, \tag{S3}$$

one can reduce the anisotropy energy $\mathcal{E}_{\text{eff}}^{\text{A}}$ to the form

$$\mathcal{E}_{\text{eff}}^{\text{A}} = -\mathcal{K}_1 m_1^2 + \mathcal{K}_2 m_2^2, \qquad \mathcal{K}_2 = \frac{1+\varkappa^2+\sigma^2-\mathcal{K}_1}{2} = \frac{2\varkappa^2}{1+\varkappa^2+\sigma^2+\mathcal{K}_1}. \tag{S4}$$

Here the coefficient $\mathcal{K}_1$ characterizes the strength of the effective easy-axis anisotropy while $\mathcal{K}_2$ gives the strength of the effective easy-surface anisotropy. The direction of effective easy axis is determined by $\boldsymbol{e}_1$ and the hard axis by $\boldsymbol{e}_2$:

$$\boldsymbol{e}_1 = \boldsymbol{e}_{\text{T}}\cos\psi + \boldsymbol{e}_{\text{B}}\sin\psi, \quad \boldsymbol{e}_3 = -\boldsymbol{e}_{\text{T}}\sin\psi + \boldsymbol{e}_{\text{B}}\cos\psi.$$

One has to note that for any finite ψ the effective anisotropy direction $\boldsymbol{e}_1$ deviates from the magnetic anisotropy direction $\boldsymbol{e}_{\text{T}}$. Note that such a deviation vanishes for wires with zero torsion ($\sigma = 0$).

Apart from effective anisotropy, the curvature and torsion show up in the effective DMI, see Eq. (S2). In the new frame of reference (ψ-frame) the effective Dzyaloshinskii energy reads[1]

$$\begin{aligned} &\mathcal{E}_{\text{ex}}^{\text{D}} = \mathcal{D}_1\left(m_2m_3' - m_3m_2'\right) + \mathcal{D}_2\left(m_1m_2' - m_2m_1'\right), \\ &\mathcal{D}_1 = 2\sigma\cos\psi + 2\varkappa\sin\psi = 2\sigma\frac{\mathcal{K}_0+\varkappa^2}{\sqrt{\mathcal{K}_0^2+\sigma^2\varkappa^2}}, \qquad \mathcal{D}_2 = 2\varkappa\cos\psi - 2\sigma\sin\psi = 2\varkappa\frac{\mathcal{K}_0-\sigma^2}{\sqrt{\mathcal{K}_0^2+\sigma^2\varkappa^2}}. \end{aligned} \tag{S5}$$

Finally we get the energy in the following form of Eq. (2) of the manuscript

$$\mathcal{E} = \underbrace{\left|\boldsymbol{m}'\right|^2}_{\text{isotropic exchange}} \underbrace{-\mathcal{K}_1 m_1^2 + \mathcal{K}_2 m_2^2}_{\text{effective anisotropy}} \underbrace{+\mathcal{D}_1\left(m_2m_3' - m_3m_2'\right) + \mathcal{D}_2\left(m_1m_2' - m_2m_1'\right)}_{\text{effective DMI}}. \tag{2}$$

[1] In the current analysis we suppose the spatio independence of the curvature and torsion (which is adequate for the helix geometry), hence $\varkappa' = \sigma' = 0$.

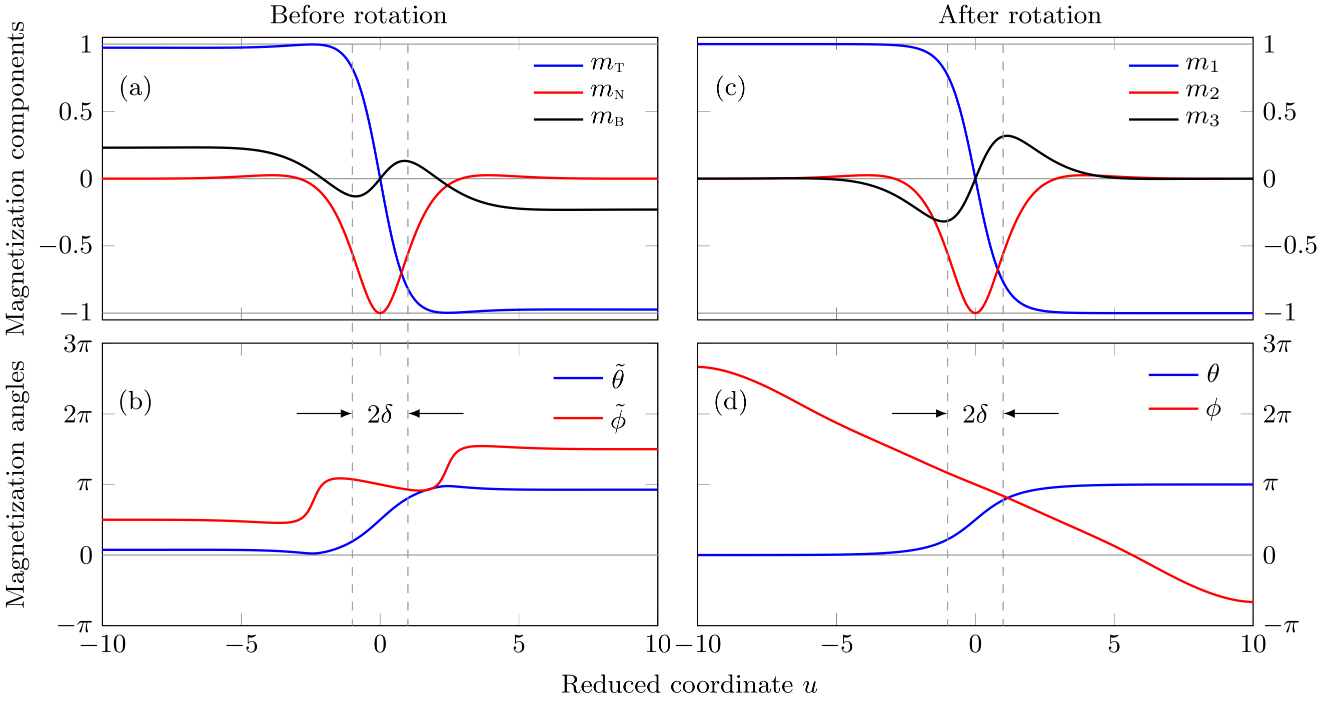

Figure S1. Comparison of the domain wall view in the TNB and the rotated reference frame (SLaSi simulations for the head-to-head domain wall): magnetization components $m_{\text{T,N,B}}$ and angles $\tilde{\theta} = \arccos m_{\text{T}}$, $\tilde{\phi} = \arctan m_{\text{B}}/m_{\text{N}}$. Right column: the same in the ψ-frame. Parameters: $\varkappa = 0.1$, $\sigma = 0.5$, $\ell = 15a$ with a being a lattice constant. Separate points are not shown due to their high density on the plots.

The dynamics of magnetization is described by the Landau–Lifshitz equations for the normalized magnetization $\boldsymbol{m}$. Using the angular parametrization,

$$\boldsymbol{m} = \cos\theta\, \boldsymbol{e}_1 + \sin\theta\cos\phi\, \boldsymbol{e}_2 + \sin\theta\sin\phi\, \boldsymbol{e}_3,$$

these equations can be derived from the Lagrangian

$$\begin{aligned} &L = K^{\text{eff}} S\ell \int \mathcal{L}\, \mathrm{d}u, \qquad \mathcal{L} = \mathcal{G} - \mathcal{E}, \qquad \mathcal{G} = -\cos\theta\dot{\phi}, \\ &\mathcal{E} = \theta'^2 + \sin^2\theta\phi'^2 - \mathcal{K}_1\cos^2\theta + \mathcal{K}_2\sin^2\theta\cos^2\phi + \mathcal{D}_1\sin^2\theta\phi' + 2\mathcal{D}_2\sin^2\theta\cos\phi\theta' \end{aligned} \tag{S6}$$

and the dissipative function

$$F = K^{\text{eff}} S\ell \int \mathcal{F}\mathrm{d}u, \qquad \mathcal{F} = \frac{\eta}{2}\left(\dot{\theta}^2 + \sin^2\theta\dot{\phi}^2\right).$$

Here and below the overdot indicates derivative with respect to the rescaled time $\bar{t} = \omega_0 t$ and $\omega_0 = \gamma_e K^{\text{eff}}/M_s$.

2 Static Domain Wall

In the case of small enough curvature ($\varkappa \ll 1$) a static domain wall in the helix wire is well described by the expression (3) of the manuscript

$$\cos\theta^{\text{dw}}(u) = -p\tanh\frac{u}{\delta}, \qquad \phi^{\text{dw}}(u) = \Phi - \Upsilon u, \tag{3}$$

where $p = \pm 1$ is a domain wall topological charge.

One can determine the magnetiochirality, *i. e.* the chirality of the magnetization structure using the Lifshitz invariant

$$\mathfrak{C} = \operatorname{sgn} \int_{-\infty}^{\infty} \left(m_2 m_3' - m_3 m_2'\right) \mathrm{d}u. \tag{S7}$$

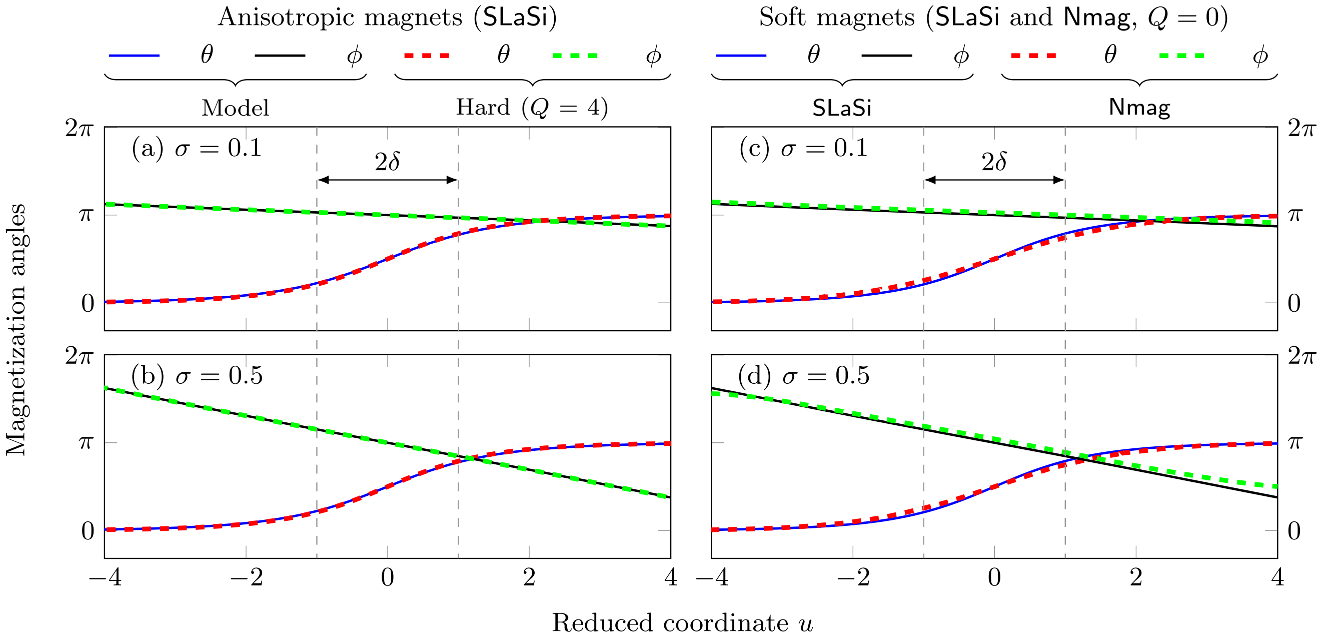

Figure S2. Influence of magnetostatics on the static domain wall: Magnetization angles in the ψ-frame for the head-to-head domain wall for $\varkappa = 0.1$ and different σ. Simulations without magnetostatics (model, solid lines) and of magnetically hard magnets ($Q = 4$, dashed lines) for $\sigma = 0.1$ (a) and $\sigma = 0.5$ (b), spin-lattice simulations in SLaSi. Simulations of magnetically soft magnets ($Q = 0$), spin-lattice simulations in SLaSi (solid lines) and micromagnetic simulations in Nmag (dashed lines) for $\sigma = 0.1$ (a) and $\sigma = 0.5$ (b). Magnetic parameters correspond to the magnetic length $\ell = 15a$. Rotation angle ψ_{sim} is determined from simulations for all curves where magnetostatics is taken into account [$|\psi - \psi_{\text{sim}}| < 0.004$, where ψ is determined by Eq. (S3)].

For the domain wall (S3) one gets $\mathfrak{C} = -\operatorname{sgn}\Upsilon$.

Let us compare the magnetization distribution in ψ-frame [Eq. (S3)] with the magnetization distribution in the TNB reference frame which are connected by the following relations:

$$m_{\text{T}} = m_1 \cos\psi - m_3 \sin\psi, \qquad m_{\text{N}} = m_2, \qquad m_{\text{B}} = m_1 \sin\psi + m_3 \cos\psi,$$

or, in the angular parametrization,

$$\cos\tilde{\theta} = m_{\text{T}} = \cos\theta\cos\psi - \sin\theta\sin\phi\sin\psi, \qquad \tan\tilde{\phi} = \frac{m_{\text{B}}}{m_{\text{N}}} = \frac{\cos\theta\sin\psi + \sin\theta\sin\phi\cos\psi}{\sin\theta\cos\phi}.$$

Comparison of the domain wall shapes in two above mentioned reference frames (magnetization components and angles) is shown in Fig. S1, obtained from SLaSi simulations,[5] c. f. Fig. 4 of the manuscript, see Methods for details. Figures S1(a) and (b) clearly pronounce that the ground state is never strictly tangential one: the component m_{B} and, therefore, the angle $\tilde{\phi}$ are nonzero far from the domain wall. In the left and the right domains the magnetization states are $\tilde{\theta} = \psi$, $\tilde{\phi} = \pi/2 \mod 2\pi$ and $\tilde{\theta} = \pi - \psi$, $\tilde{\phi} = 3\pi/2 \mod 2\pi$ respectively. Inside the domain wall a bend of the the $\tilde{\phi}(u)$ profile appears. In the rotated reference frame domain wall structure significantly simplifies: $\phi(u)$ has a shape close to linear function and m_2, m_3 components becomes localized.

Figure S2 shows a comparison of domain wall structure for different values of quality factor $Q = K/2\pi M_s^2$: $Q = 0$ and $Q = 4$ in spin-lattice simulations with micromagnetic simulations and model where dipolar interaction is replaced by easy–tangential anisotropy only, see Methods for details.

3 Effective equations of the domain wall motion under the influence of Rashba torque

In order to derive effective equations of the domain wall motion we use generalized collective coordinate q–Φ approach[6] based on the effective Lagrangian formalism. We start from the travelling wave Ansatz (see Eq. (6) of the manuscript):

$$\cos\theta^{\text{dw}}(u,\bar{t}) = -p\tanh\frac{u - q(\bar{t})}{\delta}, \qquad \phi^{\text{dw}}(u,\bar{t}) = \Phi(\bar{t}) - \Upsilon\left[u - q(\bar{t})\right]. \tag{6}$$

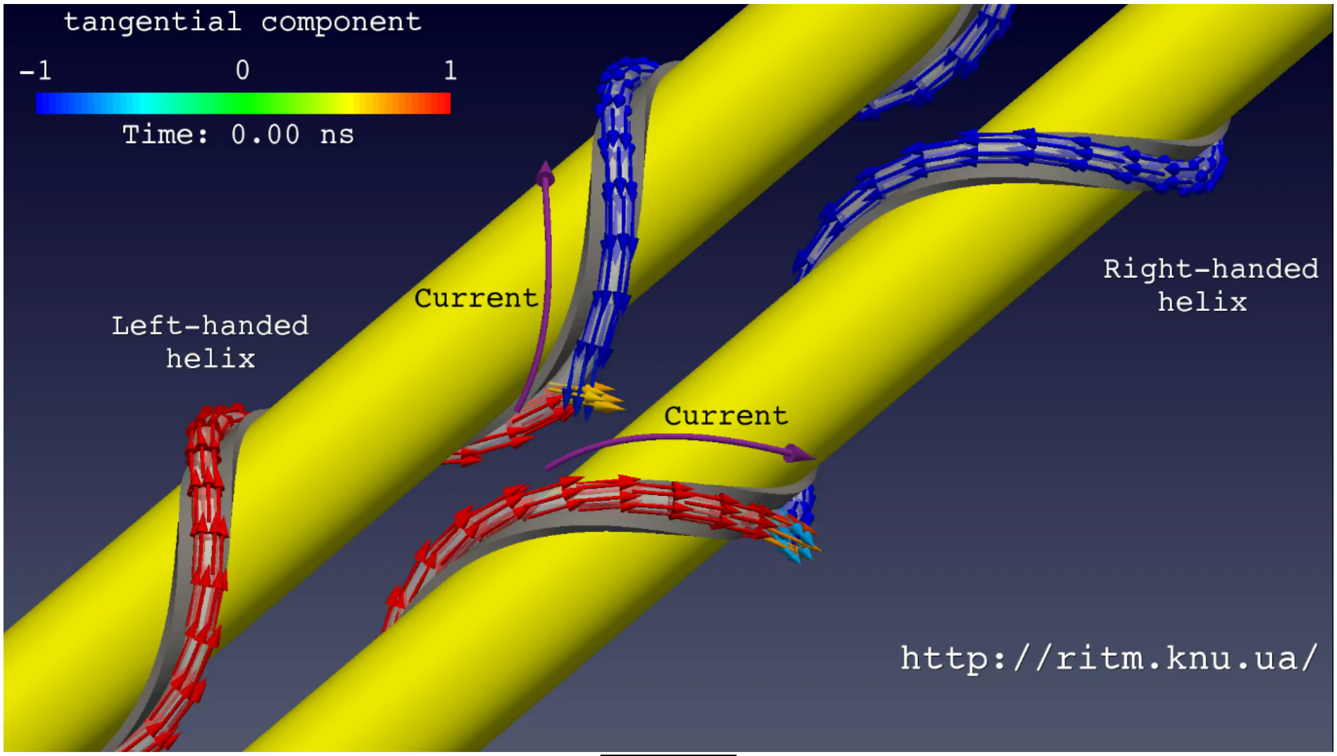

Play/Pause

Video S1. Motion of the head-to-head ($p=+1$) domain wall in helices with curvature $\varkappa=0.1$ and torsion $\sigma=\pm 0.1$ under the action of the Rashba field $h=0.02$.

One can derive the effective Lagrangian of the system by inserting this Ansatz into the full Lagrangian (S6), and calculating the integral over the dimensionless coordinate u. Then the effective Lagrangian, normalized by $KS\ell$ reads $L^{\text{eff}}=G^{\text{eff}}-E^{\text{eff}}$ with effective gyroscopical term $G^{\text{eff}}=2p\Phi\dot{q}$ and the effective energy, cf. Eq. (10) of the manuscript:

$$E^{\text{eff}}=\frac{2}{\delta}+\delta\left[2\mathcal{K}_1+2\Upsilon^2+\mathcal{K}_2\left(1+\mathcal{C}_1\cos 2\Phi\right)\right]-2\delta\mathcal{D}_1\Upsilon+p\mathcal{C}_2\mathcal{D}_2\cos\Phi-4phq\sin\psi,$$

$$\mathcal{C}_1=\frac{\pi\delta\Upsilon}{\sinh(\pi\delta\Upsilon)},\qquad \mathcal{C}_2=\frac{\pi(1+\delta^2\Upsilon^2)}{\cosh(\pi\delta\Upsilon/2)}.$$

In the same way one can derive an effective dissipative function

$$F^{\text{eff}}=\eta\left[\frac{\dot{q}^2}{\delta}+\delta\left(\dot{\Phi}+\Upsilon\dot{q}\right)^2\right].$$

From the Euler-Lagrange-Rayleigh equations (11) for the set of variables $X_i=\{q,\Phi\}$ we obtain finally

$$\dot{\Phi}\left(p+\eta\delta\Upsilon\right)+\frac{\eta}{\delta}\dot{q}\left(1+\delta^2\Upsilon^2\right)=2ph\sin\psi,\qquad \eta\delta\dot{\Phi}-\dot{q}\left(p-\eta\delta\Upsilon\right)=\mathcal{C}_1\mathcal{K}_2\delta\sin 2\Phi+\frac{p}{2}\mathcal{C}_2\mathcal{D}_2\sin\Phi. \tag{S8}$$

The effective equations of motion (S8) provide the domain wall motion with the finite velocity (see Eq. (7) of the manuscript):

$$v\equiv\frac{\mathrm{d}q}{\mathrm{d}\bar{t}}(\bar{t}\to\infty)=\frac{2ph\delta}{\eta}\cdot\frac{\sin\psi}{1+\delta^2\Upsilon^2}. \tag{7}$$

The motion of domain walls in helices with different chiralities is illustrated by Supplementary Video S1.

The stationary phase $\Phi=\text{const}$ can be found from the equation:

$$2\mathcal{C}_1\mathcal{K}_2\delta\sin 2\Phi+p\mathcal{C}_2\mathcal{D}_2\sin\Phi=-\frac{4ph\delta\sin\psi}{\eta(1+\delta^2\Upsilon^2)}\left(p-\eta\delta\Upsilon\right).$$

In the case $\varkappa, |\sigma| \ll 1$, one gets

$$\Phi \approx \Phi_0 + \frac{2h\sigma}{\pi\eta}.$$

References

1. Sheka, D. D., Kravchuk, V. P. & Gaididei, Y. Curvature effects in statics and dynamics of low dimensional magnets. *J. Phys. A: Math. Theor.* **48**, 125202; DOI:10.1088/1751-8113/48/12/125202 (2015).
2. Slastikov, V. V. & Sonnenberg, C. Reduced models for ferromagnetic nanowires. *IMA J Appl Math* **77**, 220–235; DOI:10.1093/imamat/hxr019 (2012).
3. Sheka, D. D., Kravchuk, V. P., Yershov, K. V. & Gaididei, Y. Torsion-induced effects in magnetic nanowires. *Phys. Rev. B* **92**, 054417; DOI:10.1103/PhysRevB.92.054417 (2015).
4. Yershov, K. V., Kravchuk, V. P., Sheka, D. D. & Gaididei, Y. Curvature-induced domain wall pinning. *Phys. Rev. B* **92**, 104412; DOI:10.1103/PhysRevB.92.104412 (2015).
5. `SLaSi` spin–lattice simulations package. URL http://slasi.knu.ua (Date of access:11/02/2016).
6. Kravchuk, V. P. Influence of Dzialoshinskii–Moriya interaction on static and dynamic properties of a transverse domain wall. *J. Magn. Magn. Mater.* **367**, 9; DOI:10.1016/j.jmmm.2014.04.073 (2014).
